# High-CO_2_ Treatment Prolongs the Postharvest Shelf Life of Strawberry Fruits by Reducing Decay and Cell Wall Degradation

**DOI:** 10.3390/foods10071649

**Published:** 2021-07-16

**Authors:** Hyang-Lan Eum, Seung-Hyun Han, Eun-Jin Lee

**Affiliations:** 1 Postharvest Technology Division, National Institute of Horticultural and Herbal Science, Rural Development Administration, Wanju 55365, Korea; eumhl@hanmail.net; 2 Department of Agriculture, Forestry and Bioresources, College of Agriculture and Life Sciences, Seoul National University, Seoul 08826, Korea; ejinlee73@naver.com; 3 Research Institute of Agriculture and Life Sciences, Seoul National University, Seoul 08826, Korea

**Keywords:** *Botrytis cinerea*, firmness, lipid metabolism, oligogalacturonide, pectin

## Abstract

Improved methods are needed to extend the shelf life of strawberry fruits. The objective of this study was to determine the postharvest physiological mechanism of high-CO_2_ treatment in strawberries. Harvested strawberries were stored at 10 °C after 3 h of exposure to a treatment with 30% CO_2_ or air. Pectin and gene expression levels related to cell wall degradation were measured to assess the high-CO_2_ effects on the cell wall and lipid metabolism. Strawberries subjected to high-CO_2_ treatment presented higher pectin content and firmness and lower decay than those of control fruits. Genes encoding cell wall-degrading enzymes (pectin methylesterase, polygalacturonase, and pectate lyase) were downregulated after high-CO_2_ treatment. High-CO_2_ induced the expression of oligogalacturonides, thereby conferring defense against *Botrytis cinerea* in strawberry fruits, and lowering the decay incidence at seven days after its inoculation. Our findings suggest that high-CO_2_ treatment can maintain strawberry quality by reducing decay and cell wall degradation.

## 1. Introduction

Carbon dioxide (CO_2_) plays an essential role in agriculture. During cultivation, CO_2_ enrichment is used in plant factory systems to improve yield and quality. CO_2_ also acts as a substrate for photosynthesis. In postharvest processes and cultivation, CO_2_ is used during controlled atmosphere storage or short-term high-CO_2_ treatment to maintain freshness and increase the shelf life of different fruit. However, the mechanism whereby CO_2_ maintains fruit freshness during the postharvest process remains unclear.

High-CO_2_ treatment is widely applied to reduce postharvest losses in horticultural fruits, as it effectively reduces the respiration rate and fruit decay and increases firmness [1,2,3]. In strawberry fruits, a short-term high-CO_2_ treatment of approximately 3 h increased fruit firmness with CO_2_ concentrations ranging from 10 to 100 kPa. Higher CO_2_ concentrations are usually more effective [4], but can cause physiological injuries, such as fruit discoloration and off-flavors [5].

Strawberry fruits (*Fragaria*) are easily perishable, as their fast softening reduces their postharvest and shelf life during ripening [6]. To solve this problem, high-CO_2_ treatment is used as a postharvest technology to increase firmness and reduce decay.

Plant cell walls are physically and chemically associated with firmness. The disassembly of pectin, a major component of plant cell walls, causes cell wall degradation, which leads to an increase in softening as ripening progresses [7]. Through the loss of pectic side chains during the ripening stage, increased plant cell wall porosity enhances enzymatic cell wall-degradation activity [8]. The depolymerization and solubilization of pectic substances occur because of interactions with cell wall-degrading enzymes, including polygalacturonase (PG), pectin methylesterase (PE), cellulase, pectate lyase (PL), and β-galactosidase [9,10]. PG hydrolyses α-1,4-glycosidic linkages between galacturonic acid residues, PE removes the methyl groups from the methylesterification of homogalacturonan, and cellulases break down cellulose molecules into monosaccharides or shorter polysaccharides and oligosaccharides. In addition, PL cleaves pectate groups, yielding oligosaccharides at their non-reducing ends [11]. A trend analysis of cell wall-degrading enzymes would help to better understand the relationships between changes in texture and fruit softening during ripening.

Short-term high-CO_2_ treatment induces a change in PE activity, resulting in pectin modification [12]. However, the tendency for specific PE activity after high-CO_2_ treatment has been controversial [12,13]. The increase in firmness of strawberry fruits involves the modification of pectic polymers, including a decrease in the levels of water-soluble pectins [12,14]. A specific increase in fruit firmness after high-CO_2_ treatment can be maintained for several days [14], suggesting that cell wall degradation is inhibited in fruits exposed to high-CO_2_ treatment.

In this study, gene expression levels related to fruit quality and cell wall degradation were examined after treating strawberries with a high-CO_2_ level (30%) for 3 h. Cell wall degradation in strawberry fruits was estimated using a polyuronide assay. Specific oligogalacturonides produced after high-CO_2_ treatment were also examined. A model for the mechanism of high-CO_2_ treatment is presented in this study.

## 2. Materials and Methods

### 2.1. Plant Materials, CO_2_ Treatment, and Storage

Strawberry (*Fragaria* × *ananassa* Duch. ‘Seolhyang’) fruits were harvested at the ripe stage (when they were approximately 80% red) and sorted according to their size and external skin color. Fruits with relatively uniform color and size were selected and used in this study.

Approximately 250 fruits were stored in containers at 20 °C, to which 30% CO_2_ + ambient air or ambient air (control) was provided at a rate of 10 L/min for 1 min to replace the container atmosphere; after that, the rate was adjusted to 1 L/min for 3 h. The gas concentrations were determined using a gas analyzer [(Dansensor CheckPoint, Ametek Mocon, MN, USA) every 30 min. After treatment, each lot was randomly divided into 15 groups (approximately 1 kg per group) and placed in a plastic tray without a cover. The lots were stored at 10 °C under ambient air with a relative humidity of 90 ± 5%, which was not controlled. After high-CO_2_ treatment, the fruit firmness was immediately examined, and half of the fruits (500 g) were stored at −80 °C until RNA analysis.

### 2.2. Analyses of Fruit Quality

The firmness of randomly selected fruits (n = 15) was measured using a texture analyzer (CT3 Texture Analyzer, Brookfield, MA, USA) fitted with a 5-mm cylindrical probe. Each fruit was compressed by the speed of the probe at a rate of 1 mm/s, and the maximum penetration force in Newtons (N) developed during the test was recorded.

Chromaticity was measured from the peels of 30 randomly selected fruits. The CIE L*, a*, and b* (Lab) parameters were measured using a CM-2500c spectrophotometer (Minolta, Osaka, Japan). The L* parameter represents the lightness, ranging from 0 = black to 100 = white; a* indicates a green (−) to red (+); and b* indicates a blue (−) to yellow (+) axis. Hue angle (h = tan^−1^ [b*/a*]) and chroma values (C = [a*^2^ + b*^2^]^1/2^) were calculated from a* and b* values. The hue value was determined as a color wheel, with red-purple color at 0°, yellow color at 90°, bluish-green color at 180°, and blue color at 270°, where the chroma indicates color intensity [15].

### 2.3. Respiration-Rate Measurement

The carbon dioxide concentration was measured by placing a batch of approximately 200 g of fruits in a 1 L jar sealed with a septum for 3 h at room temperature (n = 3). Gas samples were taken from each jar and the CO_2_ concentration was measured using a gas-chromatograph (YL6500, YoungLin, Anyang-si, Korea) equipped with a thermal-conductivity detector and a packed stainless steel porapak column (1.5 × 6 mm).

### 2.4. Total RNA Extraction, cDNA Synthesis, and Quantitative Real-Time PCR (qRT-PCR) Analysis

Total RNA was isolated from frozen fruit tissue during storage day using the CTAB method described by Gambino et al. [16]. The first-strand cDNA was synthesized from 5 μg total RNA using AmfiRivert Platinum cDNA Synthesis Master Mix (GenDEPOT, Katy, TX, USA). This analysis was performed using candidate primers, including eight genes (Table S1) with a 10-fold dilution solution of the cDNA product as the template. Candidate primer sequences were designed based on sequences obtained from GenBank (National Center for Biotechnology Information).

Quantitative real-time PCR was conducted in a 10 μL reaction mix equipped with CFX Connet^TM^ (Bio-Rad, Hercules, CA, USA). Each PCR mix contained 2 μL of cDNA, 4 pM of each primer, and 5 μL of 2 × Labopass^TM^ SYBR Green Q Master Mix (Cosmogenetech, Seoul, Korea). The initial denaturation step at 94 °C for 30 s was followed by 40 cycles of 95 °C for 10 s, 60 °C for 20 s, and 72 °C for 20 s, followed by a final extension at 95 °C for 10 s. Melting curve analysis was performed while increasing the temperature of each sample, ramping from 65 °C to 95 °C (increment 0.5 °C/10 s). Each reaction was performed in triplicate and the relative expression levels of control (obtained on day 1) were set to 1.

### 2.5. Cell Wall Extraction

The cell wall was extracted following a previously described method [17], with some modifications. Briefly, each tissue was immediately frozen in liquid nitrogen and stored at −80 °C until analysis. Frozen tissues were lyophilized. A total of 3 g of lyophilized fruit powder was dissolved in 100 mL of ethanol (80%, *v*/*v*) and homogenized for 1 min. Each homogenate was shaken at 250 rpm for 1 h and centrifuged (11,000× *g*) at 10 °C for 30 min. Each supernatant was discarded, and 30 mL of water was added to each residue, which was then transferred to a centrifuge tube. Each mixture was shaken at 250 rpm for 1 h and centrifuged (11,000× *g*) at 10 °C for 30 min. The supernatants were transferred to new falcon tubes and filtered through a layer of miracloth, before 30 mL of water was added to each residue. Each supernatant was transferred back to the same falcon tube by filtration through a miracloth and then mixed. Each supernatant was subjected to size-exclusion chromatography.

Each residue was washed with 100 mL of chloroform:methanol (1:1, *v*/*v*), stirred for 20 min, and then filtered through a sintered glass filter. The crude cell wall material was washed twice with 100 mL of acetone, stirred slowly, and filtered. The pellets were placed in a vacuum oven and dried at 35–40 °C for three days under P_2_O_5_. The crude cell wall material was extracted with 100 mL of 20 mM HEPES-NaOH buffer (pH 7.0) containing 1 unit/mL of α-amylase and incubated at 37 °C for 18 h. The samples were sequentially washed with 20 mM HEPES-NaOH (pH 7.0), distilled water, and acetone and then dried in a vacuum oven.

### 2.6. Size-Exclusion Chromatography

The supernatant in each falcon tube was immediately frozen in liquid nitrogen and stored at −80 °C until analysis. Frozen tissues were lyophilized. The dried samples were dissolved in 10 mL of 0.2 M ammonium acetate (pH 5.0) and mixed. Then, 1.5 mL of each mixture was transferred to a microfuge tube and centrifuged at 3000 rpm for 5 min. A total of 1 mL of each supernatant was passed through a Bio-Gel P-4 column (25 cm length × 2 cm inside diameter, Bio-Rad), and 1.1 mL fractions were collected. Size-exclusion chromatography was performed according to the method described by An et al. [18].

### 2.7. Polyuronide Assay

The supernatant (0.4 mL) was added to 40 μL of 4-M potassium sulfamate/sulfamic acid solution (pH 1.6) and 2.4 mL of sulfuric acid containing 75 mM sodium tetraborate. The mixture was heated at 100 °C for 20 min, cooled to room temperature, and then 80 μL of 0.15% (*v*/*v*) m-phenylphenol dissolved in 0.5% NaOH was added to each solution. After incubation in the dark for 30 min, absorbance was measured at 525 nm. Polyuronide content was determined by slightly modifying the method of Chatkaew and Kim [17].

### 2.8. Botrytis Cinerea Inoculation

*Botrytis cinerea* conidia collection and fruit inoculation were performed as previously described by Mengiste et al. [19]. Conidia were suspended in distilled water, and 50 µL of a spore suspension (2 × 10^5^ spores/mL) were injected into the fruits, which were subjected to a 16 h light/8 h dark cycle for three days. THunhe fruits were covered with a transparent plastic film to maintain high humidity.

### 2.9. Statistical Analysis

The experiments were designed to be completely randomized. The figures were generated using Sigma Plot software, version 10.0 (SPSS, Chicago, IL, USA). The data were statistically evaluated using IBM SPSS Statistics 21 software. An analysis of variance was performed, and the means were compared using Duncan’s test at a significance level of 0.05.

## 3. Results

### 3.1. Fruit Quality and Respiration-Rate Measurements

**Figure 1:**
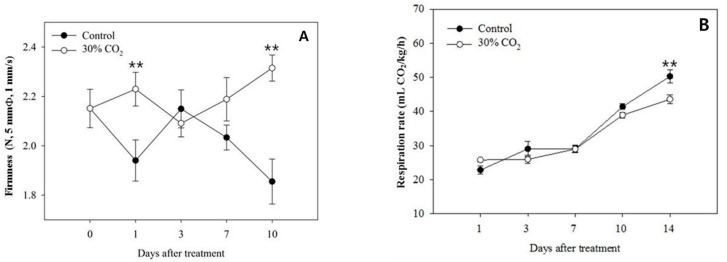
Changes in fruit firmness (**A**) and respiration rate (**B**) of strawberry fruits (Fragaria × ananassa Duch. ‘Seolhyang’) treated with 30% CO_2_ for 3 h, as well as control fruits. The data are expressed as the means ± standard errors of 15 replications. ** *p* < 0.01.

Firmness is a major index for evaluating the quality of strawberry fruits. The firmness of the strawberry fruits exposed to short-term high-CO_2_ treatment was slightly higher than that of control fruits. Ten days after treatment, while the firmness of control fruits decreased, that of high-CO_2_-treated fruits was maintained at the same levels observed on the first day after treatment (Figure 1).

The color indices (lightness, chroma, and hue angle) did not differ significantly between the high-CO_2_-treated and control fruits (Table 1). During the storage period, the CIE L* value showed a decreasing trend in both the high-CO_2_-treated and control fruits, while the CIE a* value increased. The CIE b* values of the high-CO_2_-treated and control fruits increased until the seventh day of storage and then decreased. These findings indicate that high-CO_2_ treatment did not affect the fruit skin color.

The variation in the respiration rate was not associated with high-CO_2_ treatment. Instead, it steadily increased in both the control and experimental groups for 10 days after treatment (Figure 1B). Although the control fruits showed a higher respiration rate than the high-CO_2_-treated fruits after 14 days, this difference did not affect their quality because all strawberry fruits had already decayed by then.

### 3.2. Expression Levels of Genes Associated with Cell Wall Degradation

**Figure 2:**
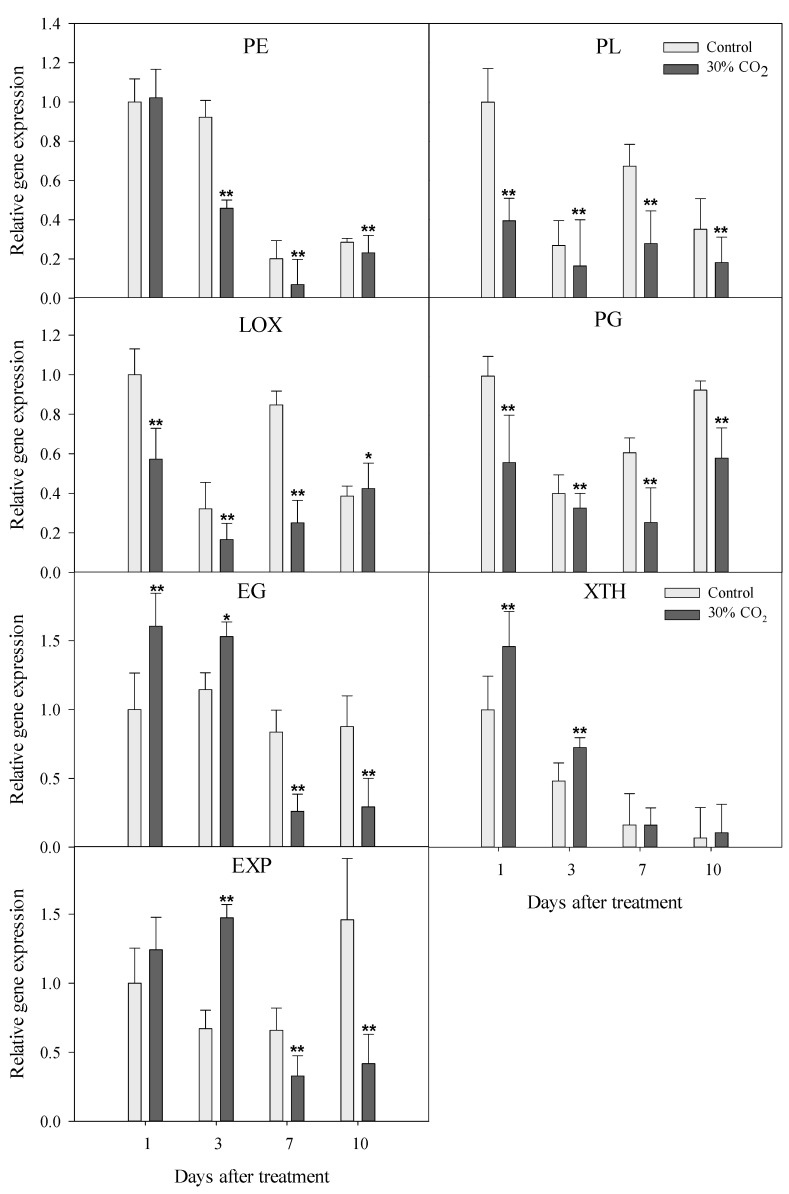
Expression levels of genes encoding cell wall-degradation enzymes in strawberry fruits treated with 30% CO_2_ for 3 h, as well as control fruits. PE, pectinmethylesterase; PL, pectate lyase; LOX, lipoxygenase; PG, polygalacturonase; EG, endo-β-1,4-glucanase; XTH, xyloglucan endotransglucosylase/hydrolase; EXP, expansin. * *p* < 0.05; ** *p* < 0.01.

The expression levels of genes encoding cell wall-degrading enzymes have been clearly related to strawberry fruit firmness [20,21]. To assess the responses to high-CO_2_ treatment, the expression levels of seven candidate genes were analyzed (Figure 2). The transcript levels of PE, PG, PL, and lipoxygenase (LOX, a lipid metabolism-associated enzyme) were relatively low in the high-CO_2_-treated fruits during storage. Regarding the hemicellulose-modifying genes, endo-β-1,4-glucanase (EG), expansin (EXP), and xyloglucan endotransglucosylase/hydrolase (XTH), higher gene expression levels were observed in high-CO_2_-treated fruits than in the control fruits three days after treatment (Figure 2). The expression levels of EG, EXP, and XTH in high-CO_2_-treated fruits were reduced to the same level or lower than those of the control fruits.

### 3.3. Polyuronides and Water-Soluble Pectin in Cell Wall Components

**Figure 3:**
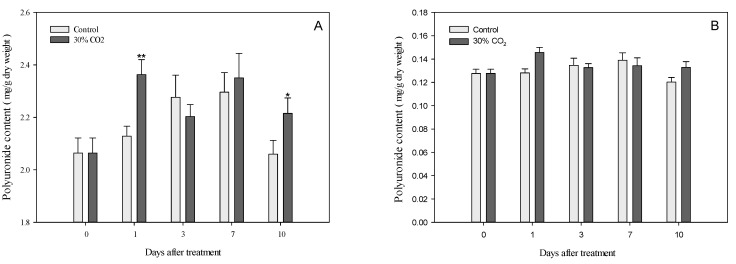
Polyuronide contents of crude cell wall material (**A**) and water-soluble pectin (**B**) from strawberry fruits treated with 30% CO_2_ for 3 h, as well as control fruits. * *p* < 0.05; ** *p* < 0.01.

The contents of polyuronides (Figure 3A) and water-soluble pectin (Figure 3B) in the crude cell wall extracts showed a diversity of pectin polymers. Significant differences in polyuronide contents were observed between the crude cell wall of the CO_2_-treated and control groups. After one day, the polyuronide contents of the crude cell wall extracts were 2.13 mg/g dry weight in control fruits and 2.36 mg/g dry weight in high-CO_2_-treated fruits. After 10 days, the polyuronide contents of the crude cell wall extracts were 2.06 mg/g dry weight in control fruits and 2.22 mg/g dry weight in high-CO_2_-treated fruits (Figure 3A).

The polyuronide content in water-soluble pectin was significantly different between the high-CO_2_-treated and control groups one day after treatment. The polyuronides contents in water-soluble pectin were 0.73 mg/g dry weight in control fruits and 0.82 mg/g dry weight in high-CO_2_-treated fruits (Figure 3B). However, no significant differences were observed in water-soluble pectin between the high-CO_2_-treated and control groups after three days.

### 3.4. Size-Exclusion Chromatography

**Figure 4:**
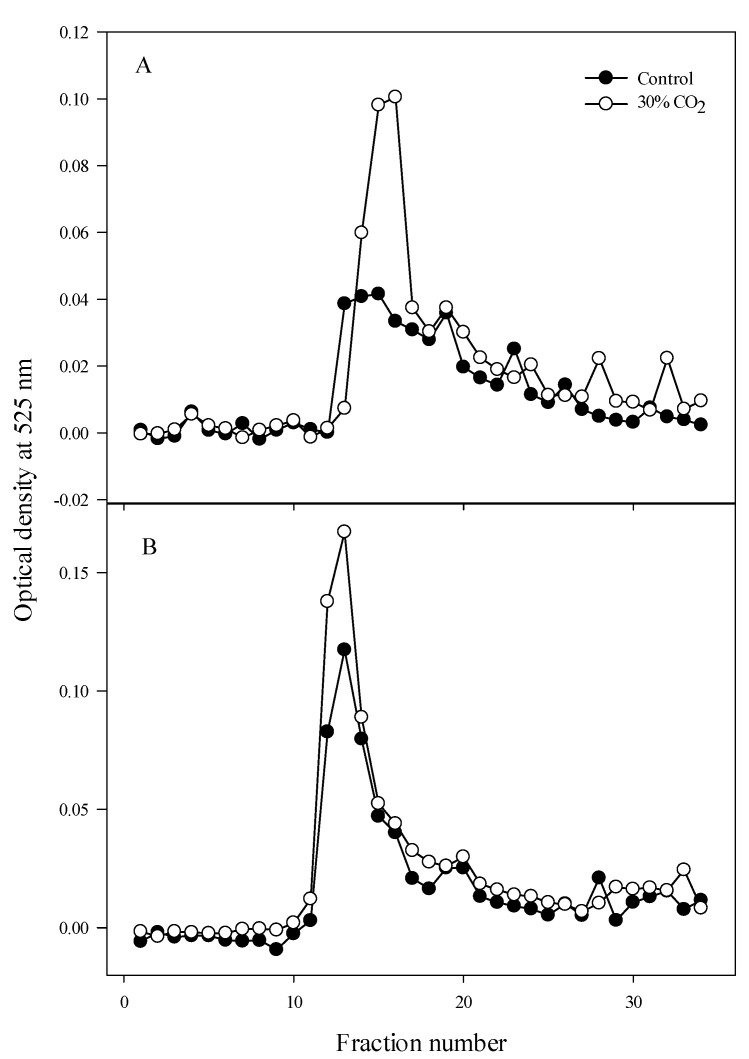
Size distribution of galacturonic acids in water-soluble pectin from strawberry fruits treated with 30% CO_2_ for 3 h, as well as control fruits. (**A**) 3 h after treatment; (**B**) 10 days after treatment.

Size-exclusion experiments were performed using column chromatography and water-soluble pectin. The galacturonic acid contents were measured in 35 different fractions (Figure 4), and showed similar trends between the high-CO_2_-treated and control fruits. A difference in the processing interval was observed between fractions 10 and 20. The galacturonic acid content was higher in high-CO_2_-treated fruits than in control fruits 3 h after treatment, as well as 10 days after treatment. After 3 h, the galacturonic acid content in fraction 15 was 0.04 mg/mL in control fruits and 0.1 mg/mL in high-CO_2_-treated fruits (Figure 4A), and after 10 days, the galacturonic acid content in fraction 12 was 0.12 mg/mL in control fruits and 0.17 mg/mL in high-CO_2_-treated fruits (Figure 4B).

### 3.5. Botrytis Cinerea Inoculation

**Figure 5:**
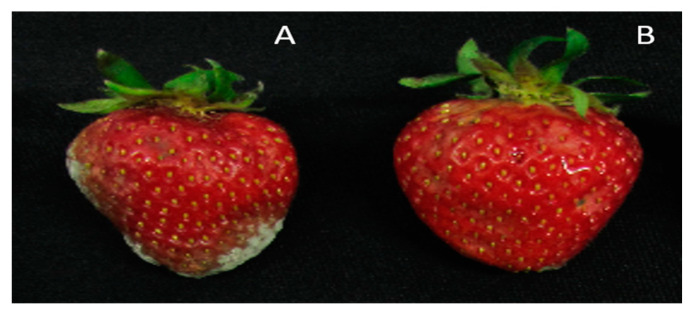
Growth of Botrytis cinerea on strawberry fruits seven days after inoculation. (**A**): control; (**B**): 30% CO_2_ treatment.

Strawberry fruits inoculated with *B. cinerea* showed typical gray mold symptoms. High-CO_2_-treated and control fruits had different lesion diameters, which ranged from 10 to 25 mm after 7 days of inoculation. Gray mold was found in both the high-CO_2_-treated and control groups, but the incidence was higher in the control group with increased lesion diameters (Figure 5). The growth of *B. cinerea* on fruits in the high-CO_2_-treated group was approximately 10% lower than that on fruits in the control group.

## 4. Discussion

Short-term high-CO_2_ treatment affected the freshness and shelf life of strawberry fruits during storage, as it showed a significant effect on firmness and decay compared to the other quality parameters (Figure 1A).

Pectin is a major component of the cell wall that directly affects the firmness of strawberry fruits [7]. Wang et al. (2014) found that an increase in firmness was associated with increased calcium ion binding to the cell wall and suppression of the activities of pectin-degrading enzymes, such as PL [3].

The structures, functions, and properties of cell walls show wide and complex biological variations [22]. The primary and secondary walls of plant cells are the major and minor sources of dietary fiber, respectively [23]. Celluloses, hemicelluloses, and pectins are the main components of primary plant cell walls that contribute to fruit texture [24]. Celluloses are unbranched, α-1,4-linked-glucan chains that aggregate by hydrogen bonding to form microfibrils approximately 3 nm in diameter. Conformationally, hemicelluloses resemble celluloses to some degree and are capable of hydrogen bonding to cellulose microfibrils, possibly in a manner similar to the aggregation of the cellulose chains themselves [9]. In most plants, the principal hemicelluloses of primary cell walls [10] are xyloglucans and glucomannans, but arabinoxylans and mixed-linkage β-glucans replace these in grasses, cereals [25], and a small number of other plants, such as palms. Pectins are homopolymers of α-1,4-linked-d-galacturonic acid with various degrees of methyl esterification on the carboxyl groups [26]. In strawberries, cell wall degradation during ripening is associated with increased pectin solubilization and hemicellulose depolymerization [27,28]. In addition, the crystallinity of the cellulose molecules remains unchanged during ripening, indicating that it may not serve a significant role in fruit softening [27].

Changes in the components of fruit cell walls are responsible for fruit softening [7]. In this study, the firmness change can be explained by the differences in the pectin contents of strawberry fruit cell walls (Figure 3A) resulting from the expression levels of genes encoding cell wall-degrading enzymes (Figure 2), and the contents of water-soluble pectin (Figure 3B).

In this study, the gene expression levels of pectin-degrading enzymes, such as PE, PG, and PL, were reduced during ripening (Figure 2). However, the gene expression levels of hemicellulose-degrading enzymes, such as EG, XTH, and EXP showed a tendency to increase until three days after high-CO_2_ treatment; after the seventh day, EG and EXP gene expression decreased in high-CO_2_-treated fruits, whereas XTH expression increased in the control group.

Expansins facilitate cell wall relaxation and affect cellulose and xyloglucan networks [29]. Brummell et al. [8] suggested that the overexpression of EXP during tomato ripening was caused by the depolymerization of hemicelluloses and pectins [8]. In maize roots, cell wall loosening is thought to result from the activities of EXP, hemicellulose, and pectin-degrading enzymes [30]. Therefore, high-CO_2_ treatment can reduce pectin degradation by suppressing the expression of genes encoding pectin-degrading and hemicellulose-degrading enzymes (especially EG), since the expression of these enzymes was not affected at the start of ripening but EG expression increased after 7 days in storage.

In our previous study, short-term exposure of strawberries to 30% CO_2_ delayed cell wall degradation by maintaining the integrity of the middle lamella [31]. The degradation of the middle lamella during ripening in various horticultural crops has been related to cell wall-degradation enzymes, such as PG and PE [7,21].

Water-soluble pectin is a general term used for small-sized pectin fragments that present water solubility [32]. The contents of water-soluble pectin were expected to differ between the two groups, based on the observed differences in firmness; however, there were no differences between the high-CO_2_-treated and control fruits (Figure 3). To determine the reason for the high water-soluble pectin content following treatment, we performed additional experiments to study the decay. The polyuronide contents (Figure 3A) of the crude cell walls observed after high-CO_2_ and control treatment were consistent with the findings related to firmness (Figure 1A).

The size-exclusion experiment revealed several pectin sizes in high-CO_2_-treated fruits (Figure 4). The existence of specific oligogalacturonides, generated by depolymerization of the parietal polysaccharides [33], might support the hypothesis that plant defense mechanisms are often produced by high-CO_2_ treatment. Oligomers of α-1,4-linked-d-GalA (OGAs) are pectin-breakdown fragments [34]. OGAs, generally referred to as endogenous elicitors, trigger defense responses as damage-associated molecular patterns. However, not all OGAs are endogenous elicitors. Specific OGAs with a degree of polymerization between 10 and 15 activate plant immune responses, such as phytoalexin accumulation [35], callose deposition, reactive oxygen species production [36], and nitric oxide production [37], by reducing decay.

Reducing decay of strawberry fruit by high CO_2_ treatment was demonstrated by *B. cinerea* inoculation experiments. As the results of this study suggest that high-CO_2_ treatment conferred a reliable decay-suppressing effect (Figure 5), and previous findings showed that OGA plays an important role in plant defense mechanisms [35,36,37], future studies should focus on clarifying the structures and functions of specific OGAs that can suppress decay.

## 5. Conclusions

High-CO_2_ treatment increased the firmness of strawberry fruit by reducing pectin decomposition and suppressing decay. Further studies could be performed to clarify whether high-CO_2_ treatment produces a specific size of OGA that elicits plant defense mechanisms. Success in that regard would make new postharvest treatments that could prolong the freshness of various horticultural crops, including strawberry fruits, feasible.

## Figures and Tables

**Table 1:** Effects of 30% CO_2_ treatment for 3 h on the peel color (L*, a*, b*), chroma, and hue angle of strawberry fruits (*Fragaria* × *ananassa* Duch. ‘Seolhyang’).

Treatment	Storage Day	CIE L*	CIE a*	CIE b*	Chroma	Hue Angle
Control	0	47.2	24.3	20.7	32.0	40.5
	1	47.5	24.2	20.8	31.9	40.6
	3	42.0	30.6	23.7	38.7	37.8
	7	40.6	30.8	26.3	40.5	40.6
	10	40.2	29.1	21.2	36.0	36.1
	14	40.5	26.9	20.7	33.9	37.6
30% CO_2_	0	47.2	24.3	20.7	32.0	40.5
	1	53.2	19.5	17.6	26.3	42.1
	3	40.0	29.6	22.5	37.2	37.2
	7	40.2	29.3	22.3	36.8	37.2
	10	41.2	28.6	21.5	35.8	36.9
	14	39.7	26.8	19.8	33.3	36.5

## Data Availability

All data are available upon reasonable request.

## Supplementary Materials

The following are available online at https://www.mdpi.com/article/10.3390/foods10071649/s1, Table S1: Candidate primers of housekeeping gene and cell wall degradation-related genes of ‘Seolhyang’ strawberry fruit.
