# Supplementary material for: High-CO2 Treatment Prolongs the Postharvest Shelf Life of Strawberry Fruits by Reducing Decay and Cell Wall Degradation
Source: Foods. 2021 Jul 16;10(7):1649. doi: 10.3390/foods10071649 (PMC8304637; doi:10.3390/foods10071649)
Supplement: Supplementary file 1 [file foods-10-01649-s001.zip › foods-1293138-supplementary.pdf]

Supplementary Information

High-CO_2 Treatment Prolongs the Postharvest Shelf Life of Strawberry Fruits by Reducing Decay and Cell Wall Degradation

Hyang-Lan Eum [1], Seung-Hyun Han [2] and Eun-Jin Lee [2,3]*

[1] Postharvest Technology Division, National Institute of Horticultural and Herbal Science, Rural Development Administration, Wanju 55365, Republic of Korea; eumhl@hanmail.net (H.L.E.)
[2] Department of Agriculture, Forestry and Bioresources, College of Agriculture and Life Sciences, Seoul National University, Seoul 08826, Republic of Korea; ejinlee73@naver.com (S.H.H.)
[3] Research Institute of Agriculture and Life Sciences, Seoul National University, Seoul 08826, Republic of Korea
* Correspondence: ejinlee3@snu.ac.kr; Tel.: +82-2-880-4565

Table S1. Candidate primers of housekeeping gene and cell wall degradation-related genes of 'Seolhyang' strawberry fruit.

Gene	Acession No.	Forward Primer (5' to 3')	Reverse Primer (5' to 3')	Size
18s	X15590	GTGCTCAAAGCAAGCCTACG	ATCTGATCGTCTTCGAGCCC	241
PE	AY324809	GAAACCTGTGGTTGCCCTCT	TTTCAAACAACACCAATGCG	256
PG	EF441274	GCAACAACGCCGAGTAGAGT	TGCAGTCGTTGTCTTTCCAAG	247
PL	U63550	ATGCTGTTGGGGCATAGTGA	TTCGCAAAACGGTTGTTAGG	236
LOX	AJ578035	CTGATGTGGCCTTCCTCAAA	TGAGGTGCTGGGATAGAGCA	284
EG	AJ006348	AACTTCGCTATCGTTCCTGCTT	GGGTTTGGATTCGGACTCAG	297
EXP	AF159563	AGAGGAGGGGGATCGAGATT	AGAACAAAGCAACTGCCCCA	271
XTH	GQ367550	CATCCCAATCCGAGTGTTCA	CTCCTTCTGATCCCACCACC	250
